# Direct Primary Care in 2015: A Survey with Selected Comparisons to 2005 Survey Data

**Published:** 2017-02-15

**Authors:** Kyle Rowe, Whitney Rowe, Josh Umbehr, Frank Dong, Elizabeth Ablah

**Affiliations:** 1 Department of Internal Medicine, University of Kansas School of Medicine-Wichita, KS; 2 Department of Family and Community Medicine, Family Medicine Residency Program at Wesley Medical Center, University of Kansas School of Medicine-Wichita, KS; 3 Atlas MD Concierge Family Practice, Wichita, KS; 4 Western University of Health Sciences, Pomona, CA; 5 Department of Preventive Medicine and Public Health, University of Kansas School of Medicine-Wichita, KS

**Keywords:** primary health care, concierge medicine, retainer medicine, delivery of health care

## Abstract

**Introduction:**

Direct primary care (DPC), a fee for membership type of practice, is an evolving innovative primary care delivery model. Little is known about current membership fees, insurance billing status, physician training, and patient panel size in DPC practices. This study aimed to obtain current data for these variables, as well as additional demographic and financial indicators, and relate the findings to the Healthy People 2020 goals. It was predicted that DPC practices would (1) submit fewer claims to insurance, (2) have decreased membership fees, (3) be primarily family medicine trained, and (4) have increased the projected patient panel size since 2005.

**Methods:**

An electronic survey was sent to DPC practices (n = 65) requesting location, membership fees, projected patient panel size, insurance billing status, training, and other demographic and financial indicators. Data were aggregated, reported anonymously, and compared to two prior characterizations of DPC practices done in 2005.

**Results:**

Thirty-eight of 65 (59%) practices responded to the 2015 survey. The majority of respondents (84%) reported using an EMR, offering physician email access (82%), 24-hour access (76%), same day appointments (92%), and wholesale labs (74%). Few respondents offered inpatient care (16%), obstetrics (3%), or financial/insurance consultant services. Eighty-eight percent (88%) of practices reported annual individual adult membership rates between $500 and $1,499, decreased from 2005 where 81% reported greater than a $1,500 annual fee. The proportion of practices who submit bills to insurance decreased from 75% in 2005 to 11% in 2015. Fifty-six percent (56%) of practices reported projected patient panel size to be greater than 600, increased from 40% in 2005. Family medicine physicians represented 87% of respondents, markedly different from 2005 when 62 – 77% of DPC respondents were general internal medicine physicians.

**Conclusions:**

Most DPC practices no longer submit to insurance and are family medicine trained. Compared with the previous sampling, DPC practices report decreased membership fees and increased projected panel size. These trends may signify the DPC movement’s growth in application and scope.

## Introduction

Direct primary care (DPC), also known as “concierge medicine,” has been increasing in popularity since the early 2000s.^1^–^3^ The practice discipline is based on the premise that the development of a high quality patient-physician relationship is enhanced in an environment that provides unrestricted access, innovative and open communication, and increased face-to-face time. Patients pay a practice determined membership fee, at varying intervals ranging from monthly to annually, in exchange for a variety of included amenities and services which are intended to support this premise.^4^ Patients are attracted to this model for the simplicity, and the quality of the relationship they potentially can build with their physicians.^1^ The patient’s preventive care becomes the primary focus. It follows that there should be a decreased disease burden, decreased utilization of acute care, inpatient, and specialist services, thus decreased health care cost. Decreased numbers of emergency department visits, as well as decreased inpatient admissions, can occur among Medicare beneficiaries utilizing a direct primary care model.^5^

In October 2014, the Centers for Medicare and Medicaid Services (CMS) dedicated $840 million in grant support for primary care innovation, one component of which was specified as initiatives developing and testing new payment and service delivery models.^6^ This sizable commitment may aid in achieving the Healthy People 2020 goals of increased supply, access, and utilization of primary care services.^7^ In an environment of high primary care burnout,^8^ innovative models that promote greater balance between work and home-life, at similar levels of compensation, will become more needed to increase the supply of primary care physicians. Additional study in the DPC style of practice will facilitate further innovations toward these ends and will aid emerging physicians’ choices of specialty and practice models. In 2005, previous researchers analyzed multiple components of DPC practices including the membership fees, insurance billing status, projected panel size, and specialty.^9^,^10^ Little is known about the change in the aforementioned practice characteristics from 2005 to 2015. These specific points are relevant to understanding the growth and development of DPC. Therefore, this survey aimed to obtain these current data points, obtain additional demographic and financial indicators, and relate the findings to the Healthy People 2020 goals. It was predicted that DPC practices would (1) submit fewer claims to insurance, (2) have decreased membership fees, and (3) have increased the projected patient panel size since the last evaluation.

## Methods

### Participants

This study was deemed “non-human subjects” research by the Institutional Review Board at the University of Kansas School of Medicine-Wichita. Potential practices to survey were identified using the Google^™^ search engine for the terms “direct primary care” and “concierge medicine.” Practices that clearly self-identified with these labels were chosen from the top 100 search results. If no practice email was readily available on the website, a phone call was made to request participation and contact information. Additional participants were identified using snowball sampling, wherein respondents suggested other DPC practices to receive the survey.

### Instruments

Data were collected using a survey instrument distributed via the Survey Monkey^®^ online platform. The survey instrument was developed solely for this study, based on the intent to compare data to previous characterizations. Additional items were added to obtain practice demographics, financial characteristics, and to expand on the possible amenities offered as described below. The continuous variables included years in practice, the number of physicians in the practice, number of staff members, and membership fees. Physician salary, work hours, and patient panel size were collected using interval values. Physician salary and patient panel size were collected in current and projected forms, with ‘projected’ being defined as the desired end point for the practice, rather than a distinct time period. Discrete variables included state of practice, practice setting (rural or urban), residency training, acceptance of Medicare patients, size of practice, and the presence or absence of numerous services and amenities including: electronic medical record (EMR), patient portal, physician email access, social networking (i.e., Twitter, Facebook), financial or insurance patient consultant, 24-hour physician access, same day appointments, house calls, inpatient care, obstetric care, wholesale labs, wholesale medications, wholesale imaging, employer group contracts, and immunizations. Additionally, there were two free text fields for any additional comments, as well as for referral contact information for other DPC practices.

### Procedures

Sixty-five practices were identified. They were sent an email containing a link to the online survey that requested their participation in the study. In the initial email, they were assured of anonymous data reporting. A reminder email was sent four weeks later. There was a subsequent four-week interval until data were collected for analysis.

### Analysis

Data were collected and analyzed using the SAS software for Windows version 9.3 (Cary, NC). Descriptive statistics were presented as frequencies and proportions for categorical variables. A one-sided binomial proportion comparison was conducted using PROC FREQ. The 2015 data were compared to the corresponding 2005 proportions. Membership fees, reported as continuous variables, were aggregated into interval ranges allowing comparison to the prior studies. Data are presented as a percentage of respondents reporting.

## Results

### 2015 Survey Results

#### Practice demographics, physician salaries and work hours

**Figure 1:**
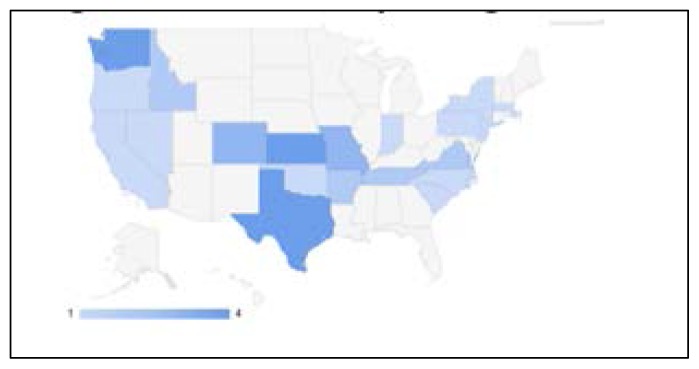
States of responding practices*.* Darker color indicates higher number of responses in state.

**Figure 2:**
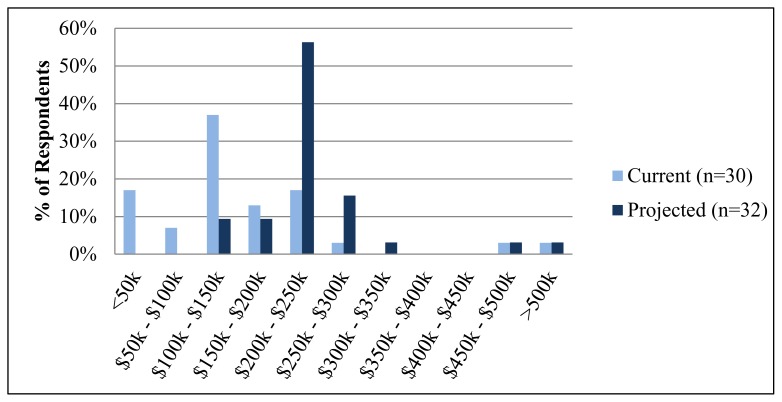
Current and projected incomes for DPC practices from 2015 survey results.

Of the 65 direct primary practices sent an invitation to participate in the survey, responses were received from 38 (59% response rate), representing 20 different states (Figure 1). The majority of respondents (74%) reported physicians spend fewer than 50 hours per week devoted to patient care and practice management (Table 1). Most (72%) reported projected physician incomes between $200,000 and $300,000, and half reported current physician incomes between $100,000 and $200,000 (Figure 2). Two-thirds (65%) of respondents reported their practice as urban, and one-third (35%) as rural. Sixty-one percent (61%) of respondents reported having transitioned from a traditional practice and 39% were de novo practices. Most (70%) respondents reported being in practice for one year or less, and reported having fewer than six employees (94%).

**Figure 3:**
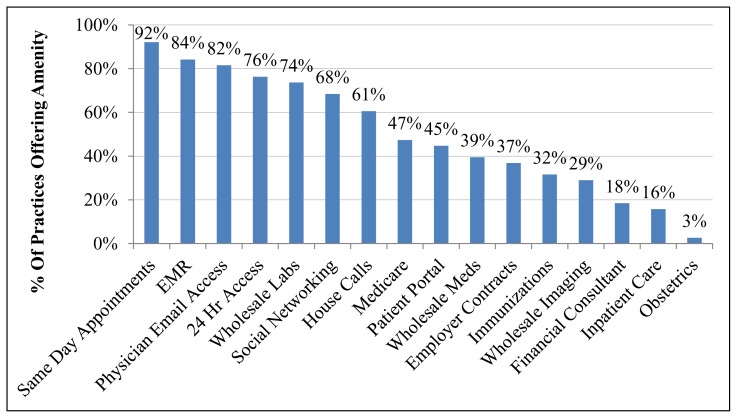
Percent of practices reporting various amenities in 2015.

Practice amenities are presented in Figure 3. The majority of respondents (84%) reported using an EMR, offering physician email access (82%), 24-hour access (76%), same day appointments (92%), and wholesale labs (74%). Few respondents offered inpatient care (16%), obstetrics (3%), or financial/insurance consultant services (18%). Survey data regarding these points were not available from 2005 for full comparison.

## Selected Comparisons to 2005

**Figure 4:**
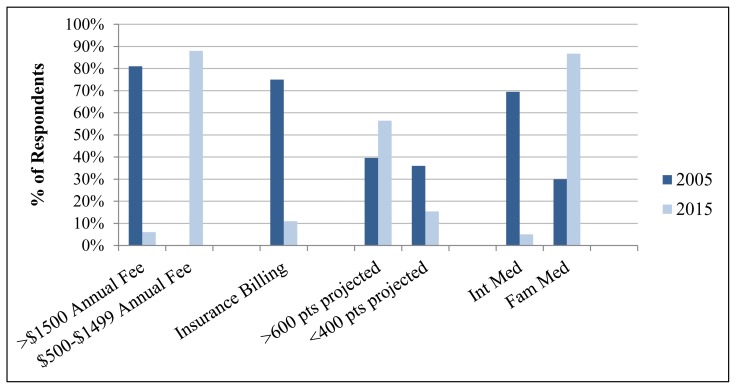
Selected comparisons to 2005 survey results. Note that no tests for statistical significance were used.

Specialties, membership fees, insurance billing, and projected panel size were compared between 2005 and 2015. The results are presented in Figure 4. In 2015, most respondents (87%) were family medicine physicians; 5% were internal medicine physicians, with the remainder from pediatrics or internal medicine/pediatrics. This was a statistically significant reduction from the 62 – 77% of DPC practices reported as internal medicine training in 2005 (p < 0.0001, compared to the 62% to be conservative). Second, the majority (88%) of respondents reported annual individual adult membership rates of $500 – $1,499, a significant increase from 2005 where 19% reported between $500 and $1,499 (p < 0.0001). Third, few practices (11%) reported submitting bills to insurance in 2015, a statistically significant decrease from the 75% in 2005 (p < 0.0001). Last, fifty-six percent (56%) of practices reported projected patient panel size ≥ 600 in 2015, a statically significant increase from 40% in 2005 (p = 0.0274).

## Discussion

DPC, once known as a model focused on providing care for the wealthy, appears to be undergoing a transformation into a model that is more accessible to the general population. Although 58% of the practices surveyed reported current physician patient panel sizes of fewer than 400 patients (Figure 2), a large number of this study’s respondents reported being in practice for one year or less, and the intention to grow was present. This assumption was based on the projected patient panel size, a number that reflects the eventual goals DPC providers have for their practice and community. Providing more affordable care to an increased number of patients than previously suggested expands the reach and impact that DPC may have on communities at large, compatible with the Healthy People 2020 goal of increasing patient access to primary care.

Additionally, as many as one-third of primary care physicians may have a high level of burnout, making the delivery of quality care highly dependent on physician and practice environment.8,11 One identified factor of burnout is patient panel size.^8^ The increase in projected patient panel size of DPC practices, though well below that of traditional practice models,^12^ could suggest that DPC physicians are improving the balance between meeting needs of the community and their lifestyle. Additional factors addressed in this survey that are known to affect physician satisfaction and specialty choice include reduced paperwork^13^ resulting from decreased insurance billing, and similar salaries and work hours compared to traditional primary care physicians.^14^ Practicing in a model such as this may attract more emerging physicians to enter primary care, contributing to the Healthy People 2020 goal of increasing primary care supply.

### Limitations

The shift in training of DPC physicians from mainly internal medicine (62 – 77%) in 2005 to family medicine (86%) is difficult to interpret and is a potential limitation of the study, questioning the sampling of practices and generalizability of the results. This study’s result, however, is representative of practices distinctly self-identifying as DPC. Dissatisfaction in primary care has led to a decrease in general practice internists,^15^ with more graduating residents entering hospital or specialty medicine. This change was likely mirrored in DPC and may account for these findings. A more thorough evaluation of physician attitudes towards DPC is warranted, particularly comparing differences between the primary care specialties.

Another limitation of this survey involved the method of determining membership fees. Fee schedules were often complex and influenced by multiple factors including age of member, number of dependents, and employment status. However, our comparison was based on the average individual adult fee, which is consistent with prior studies. Further research to analyze the variety of fee schedules in DPC practices is merited.

### Implications

Of the 38 respondents, only one provided obstetrical care, and six provided inpatient care. These results highlighted that DPC, though promoting preventive medicine, chronic disease management, and accessible acute care for minor illness, did not always provide comprehensive care. Collaboration with hospital networks, insurance providers, and specialty services is a necessity for high quality comprehensive healthcare. Research defining these relationships would help to understand the role of DPC better within the medical community at large. Furthermore, little is known about DPC within communities dominated by larger integrated systems, such as Intermountain Healthcare or Kaiser Permanente, that share many principles of DPC, including membership-based comprehensive, accessible, and patient-centered care. DPC potentially could function as a pathfinder and catalyst for change toward a higher level of healthcare integration and cooperation, especially in communities strongly rooted in fee-for-service systems. Research directed toward this question would be valuable.

## Conclusions

Compared to 2005 survey data, membership fees for direct primary care have decreased significantly and projections of patient panel size have increased significantly, suggesting that the model is in motion towards more generalizable application. The rate of insurance billing has decreased significantly, and the model is now predominantly family medicine. DPC may serve as a viable model to support the primary care goals set by Healthy People 2020.

## Figures and Tables

**Table 1:** Practice and Physician characteristics from 2015 survey.

Average Weekly Work Hours	% of Respondents
<40	26.3%
41–50	47.4%
51–60	15.8%
61–70	5.3%
71–80	2.6%

**Rural Vs Urban**	
Rural	35.1%
Urban	64.9%

**Transitioned DPC vs De Novo DPC**	
De Novo DPC	38.9%
Transitioned DPC	61.1%

**Number of Staff in Practice**	
One	33.3%
Two	19.4%
Three	13.9%
Four	11.1%
Five	11.1%
Six	5.6%
>20	5.6%
